# SARS-CoV-2 diagnosis: a single-centre experience

**DOI:** 10.25122/jml-2021-0064

**Published:** 2021

**Authors:** Ion Maruntelu, Andreea Mirela Caragea, Maria Tizu, Ileana Constantinescu

**Affiliations:** 1. Department of Immunology and Immunology of Transplant, Carol Davila University of Medicine and Pharmacy, Bucharest, Romania; 2. Centre of Immunogenetics and Virology, Fundeni Clinical Institute, Bucharest, Romania

**Keywords:** COVID-19, SARS-CoV-2, RT-PCR, anti nucleocapside, anti-protein S, patients and healthcare workers, Ig – immunoglobulin, RNA – ribonucleic acid, RT-PCR – reverse transcription-polymerase chain reaction, WHO – World Health Organization

## Abstract

The coronavirus disease 2019 (COVID-19) was declared a pandemic by the World Health Organization (WHO) on the 11^th^ of March 2020. In Romania, there have been 983,217 confirmed cases and 24,386 deaths. We aim to show our experience at the Fundeni Clinical Institute in the diagnosis of Severe Acute Respiratory Syndrome Coronavirus-2 (SARS-CoV-2) infection in both patients and health care personnel. Swab samples were collected for extraction of the SARS-CoV-2 RNA from 29380 patients and health care personnel. We have combined three real-time reverse transcription-polymerase chain reaction (RT-PCR) assays for the qualitative detection of SARS-CoV-2. Also, the presence of IgG against SARS-CoV-2 nucleoprotein was analyzed in 1068 patients and clinical staff using the chemiluminescence method. Other 50 people were screened post-vaccination for the presence of SARS-CoV-2 antibodies against the spike (S) protein, using the chemiluminescence method as well. The majority of confirmed cases were in adults, 71.3% of cases being registered in people aged 30-69 years. Most patients diagnosed with SARS-CoV-2 infection (83%) were admitted to the gastroenterology, hematology, and surgery wards. Our study showed that one-third of people developed antibodies against the nucleocapsid of SARS-CoV-2. SARS-CoV-2 IgG seroprevalence does not vary by gender or age. Also, we noticed the presence of antibodies against the SARS-CoV-2 spike protein in all 50 people post-vaccination that were tested two weeks after the second dose. Due to the increasing number of infected patients with SARS-CoV-2, the new coronavirus pandemic involves a sustained testing effort for an accurate virological diagnosis in both direct and indirect diagnosis.

## Introduction

In December 2019, many people with acute respiratory syndrome were identified in Wuhan, a city from Hubei, China [1]. In the following months, a similar acute respiratory disease has also been reported worldwide [2]. The World Health Organization (WHO) named the disease coronavirus disease 2019 (COVID-19) and declared it a pandemic in the next few months [3]. The International Committee on Taxonomy of Viruses renamed 2019-nCoV to Severe Acute Respiratory Syndrome Coronavirus-2 (SARS-CoV-2). This virus was detected in different secretions from infected cases (i.e., throat swabs, conjunctival swabs, sputum) [4]. Thus, the main mode of transmission is from one person to another through different types of secretions. People of all ages could be infected, but the risk for complications increases with age [8] and immunological status. Around the world, 71.3 million people have been infected with SARS-CoV-2, and more than 1.6 million people have died from the disease, according to the WHO [5]. At the time of writing, there have been 983,217 confirmed cases of COVID-19 and 24,386 deaths in Romania [5].

Full-genome sequencing and phylogenetic analysis indicated that SARS-CoV-2 is a member of the coronavirus family. Determination of SARS-CoV-2 partial genome sequence allowed the development of real-time reverse transcriptase-polymerase chain reaction (RT-PCR) methods for the detection of SARS-CoV-2 [6, 7]. RT-PCR tests were based on RNA-dependent RNA polymerase (RdRp), ORF1ab, E, N, and S genes of the SARS-CoV-2 genome [8–11].

We aimed to share our experience at the Fundeni Clinical Institute in the diagnosis of SARS-CoV-2 infection in both patients and health care personnel and to reveal the specific immunization.

## Material and Methods

This study was carried out at the Fundeni Clinical Institute, Bucharest, Romania. Both nasal and oropharyngeal specimens were collected from patients and health care workers, and electronic medical records provided various epidemiological and clinical data.

### Real-time reverse transcription-polymerase chain reaction assay for COVID-19

Swab samples were collected for the extraction of SARS-CoV-2 RNA from 23338 patients and clinical staff. We have approached direct virological diagnosis using three real-time RT-PCR assays for the qualitative detection of SARS-CoV-2 (Bosphore, Anatolia Geneworks, Turkey; Seegene, South Korea; GeneXpert, Cepheid, United States of America). These three assays were used to validate the methods (interassay validation) and also to cover the emergencies for the transplantation procedure. Bosphore Anatolia Geneworks products targeted the orf1 ab genome region, N gene (added in the last version of the kit) and E gene. Seegene products targeted the N gene, RNA-dependent RNA polymerase –RdRp and E gene, while GeneXpert SARS-CoV-2 tests detected the E and N2 genes in less than an hour. The GeneXpert SARS-CoV-2 test was used to screen patients before the transplantation procedure. The RT-PCR tests were settled according to the manuscript instructions. Also, the presence of IgG against SARS-CoV-2 nucleoprotein was analyzed in 1068 patients and clinical staff using the chemiluminescence method (SARS-CoV-2 IgG assay, Abbott, Illinois, United States of America). We have started to investigate the anti-spike antibody onset after vaccination with Pfizer, AstraZeneca, and Moderna vaccines.

### Statistical analysis

Statistical analysis was done using the Statistical Package for the Social Sciences (SPSS) version 13.0 software (SPSS Inc). The categorical variables are summarized numbers and percentages.

## Results

We have included in our study all the patients that were tested for before their admission to the hospital. Also, 2382 individuals that are part of the medical staff of the Fundeni Clinical Institute were analyzed. All participants were tested for the SARS-CoV-2 infection. The median age of the patients was 55.72 years, and 52.21% of them were men. The median age was 46 years for the clinical staff, and 70.44% of them were women. Patients from the entire country usually come to be treated in our Institute, but most of the patients were from Bucharest-Ilfov (35.5%). The highest number of SARS-CoV-2 tests was requested from the gastroenterology, hematology, oncology, neurology, and urology wards, which represented 75.49% of all tests (Table 1). Regarding the patients, the positive rate of the RT-PCR tests was 7.23%. All the patients positive for the SARS-CoV-2 infection had one or more comorbidities (diabetes, obesity and others) and were aged between 1 and 101 years old. The biggest incidence of positive cases (72.42%) was seen in individuals aged 39–74 years.

**Table 1.** Positive and negative RT-PCR tests in the most important wards of the Fundeni Clinical Institute.

Row Labels	Negative RT-PCR	Positive RT-PCR	Sum of grand total RT-PCR
**Gastroenterology**	2345	73	2418
**General Surgery**	989	58	1047
**Hem cell stem transplantation**	102	16	118
**Hematology**	1896	161	2057
**Intensive Care Unit**	63	18	81
**Internal Medicine**	1267	72	1339
**Kidney transplantation**	86	6	92
**Nephrology**	844	46	890
**Neurology**	1177	77	1254
**Oncology**	1591	119	1710
**Pediatric**	343	18	361
**Urology**	2316	74	2390

The highest number of positive cases was noticed in patients admitted to the hematology and oncology departments, and we have correlated this to their secondary immunodeficiencies (Table 1).

Three hundred eighty-nine healthy individuals who were part of the medical staff were also positive for the SARS-CoV-2 infection. Most of the infected medical workers (212) came from the intensive care unit, general surgery, gastroenterology, and hematology departments. The rate of positive RT-PCR results was higher in the case of female health care workers (n=293) than males (n=96).

Also, 1068 patients and health care personnel were screened for the presence of SARS-CoV-2 IgG anti-nucleocapsid antibodies using the chemiluminescence method (Table 2). The test for SARS-CoV-2 IgG antibodies revealed that 253 patients and 157 health workers of the Institute developed IgG antibodies against the virus. Other 50 people (patients and medical staff) were screened post-vaccination for the presence of SARS-CoV-2 antibodies against the spike (S) protein. All of them developed antibodies against SARS-CoV-2 spike protein.

**Table 2.** Results of the anti-SARS-CoV-2 IgG tests.

Anti-SARS-CoV-2 IgG	Negative RT-PCR	Positive RT-PCR	No previous RT-PCR	Grand total
**Negative**	255	38	365	658
**Positive**	117	78	215	410
**Grand total**	372	116	580	1068

## Discussion

SARS-CoV-2 targets alveolar epithelial cells and enterocytes, leading to Covid-19 [12, 13]. The disease is predominantly characterized by respiratory symptoms and may progress to bilateral interstitial pneumonia, respiratory failure, or acute respiratory distress syndrome (ARDS) [14]. According to a WHO report from March 2021, there have been 983,217 confirmed cases of COVID-19 and 24,386 deaths in Romania [5].

At the Fundeni Clinical Institute, a significant number of RT-PCR tests for the SARS-CoV-2 infection were performed for both admitted patients and medical personnel. Our experience in the interpretation of RT-PCR results revealed at least four possible profiles. The presence of the E gene without Orf1ab/RdRp and N genes indicated a possible infection with coronaviruses and retesting is advised. The presence of the E, Orf1ab/RdRp and N genes at a cycle threshold (Ct) value of less than 30 indicates an infection with the SARS-CoV-2 virus. If the Ct value is higher than 30, RT-PCR should be completed with SARS-CoV-2 IgG screening in order to check if the individual was immunized. Immunized patients with positive RT-PCR tests were not considered to be an epidemiological risk. When only a specific SARS-CoV-2 gene was identified (at a Ct value less than 30), infection with SARS-CoV-2 was confirmed. Otherwise, the RT-PCR test was repeated.

We have noticed a slight predominance of the female gender (59% of the total cases, M/F ratio – 0.84) in our positive results. The majority of confirmed cases were in adults, 71.3% of cases being registered in people aged 30–69 years. Most patients diagnosed with SARS-CoV-2 (83%) were admitted to the gastroenterology, hematology, and surgery wards. The majority of the patients were immunocompromised. Our findings are similar to Chen *et al.*, who concluded that the SARS-CoV-2 infection seems to affect older men with different comorbidities (i.e., diabetes mellitus and malignancies) [1]. Guo *et al.* showed that the rate of SARS-CoV-2 positive cases was higher in intensive care units (ICU) compared to the other wards [15]. Anti-SARS-CoV-2 IgG anti-nucleocapsid antibodies were identified in 38.38% of cases (from 1068 tests). One hundred fifty-seven of the total confirmed RT-PCR cases were registered among the medical staff, and 253 patients were found to be immunized against COVID-19. Our results revealed that a relevant number of people working in the ICU got immunized against SARS-CoV-2 with no signs and symptoms of the disease. One-third of the tested people developed anti-nucleocapsid antibodies against SARS-CoV-2. SARS-CoV-2 IgG seroprevalence was not changed by gender or age. Differences were revealed in immunocompromised people. Concordant results were published by colleagues from China and Spain [16]. In the United States, researchers found that a large number of the population appeared to be unexposed to COVID-19, even in areas where the new coronavirus has become widespread [16].

These findings suggest that complete immunization could not be possible. Transmission pathways are not fully understood until now. As an RNA virus, SARS-CoV-2 has a high rate of mutations that occur during the multiplication cycle of the virus resulting in new viral strains. Therefore, the emergence of a new SARS-CoV-2 strain is conditioned by its ability to multiply and disseminate to susceptible organisms [17]. In order to limit the spread of the epidemic, emergency epidemiological measures (isolation, quarantine, restriction of movement and human contact) should be kept. From December until March 2021, in Romania, only messenger RNA-based vaccines were used. The messenger RNA enters the human cells, where it transmits the necessary information for the production of its viral proteins, against which, subsequently, antibodies will develop. In our center, we noticed the presence of antibodies against SARS-CoV-2 S protein in all 50 people post-vaccination that were tested by the chemiluminescence method (SARS-CoV-2 IgG assay, Abbott, Illinois, United States of America) two weeks after the second dose. Our preliminary results showed arbitrary units (AU) values higher than 3000 in all these people. During the studied time, we did not have reinfected patients pre- or post-vaccination.

However, we have noticed that anti-nucleocapsid IgG antibodies decreased over time. Unfortunately, anti-spike IgG antibody levels detected after immunization also decreased over time. We are wondering what happens with these antibodies by the end of the year. As for influenza viruses, we think that immunization against SARS-CoV-2 should be done periodically to have a certain protective level of specific antibodies.

## Conclusions

Due to the increasing number of patients infected with SARS-CoV-2, the new coronavirus pandemic involves a sustained testing effort for an accurate virological diagnosis in both aspects of direct and indirect diagnosis. Upon admission, all patients must be tested for COVID-19. If suspicious (fever, respiratory symptoms), contact precautions must be carried out until a negative PCR result is obtained. This will prevent the hospital from becoming a source of SARS-CoV-2 infection which could spread among the patients and medical staff. Patients aged 30–69 with secondary immunodeficiency were at significant risk of getting infected with the new coronavirus.

Anti-nucleocapsid antibodies revealed after natural infection or anti-spike antibodies that appear post-vaccination do not mean that patients and health care workers could not get infected with the new coronavirus again. We have to keep in mind that SARS-CoV-2 is an RNA virus with a high mutation rate within the genome. Therefore, it is not yet clear how long and how efficient specific anti-SARS-CoV-2 antibodies are. More accurate studies are needed to understand the dynamics of the immune response and how both innate and adaptive immunity fights against COVID-19.

## Acknowledgments

### Data availability

The data that supports the findings of this study are available from the electronic medical records of our institution and can only be accessed with approval of the system manager.

### Conflict of interest

The authors declare that there is no conflict of interest.
